# Review of Non-Invasive Fetal Electrocardiography Monitoring Techniques

**DOI:** 10.3390/s25051412

**Published:** 2025-02-26

**Authors:** Xiongjun Li, Jingyu Wan, Xiaobo Peng

**Affiliations:** 1 College of Physics and Optoelectronic Engineering, Shenzhen University, Shenzhen 518060, China; 2300453025@email.szu.edu.cn; 2 College of Mechatronics and Control Engineering, Shenzhen University, Shenzhen 518060, China

**Keywords:** electrocardiography, fetal electrocardiography, non-invasive fetal monitoring, fetal heart rate, cardiac anomaly classification, deep learning

## Abstract

Non-invasive fetal electrocardiography (NIFECG), an emerging technology for fetal health monitoring, has garnered significant attention in recent years. It is considered a promising alternative to traditional Doppler ultrasound methods and has the potential to become the standard approach for fetal monitoring. This paper provides a comprehensive review of the latest advancements in NIFECG technology, including signal acquisition, signal preprocessing, fetal electrocardiogram extraction, and fetal cardiac anomaly classification. Furthermore, the characteristics and limitations of existing NIFECG datasets are analyzed, and improvement suggestions are proposed. Future research directions for NIFECG technology are discussed, with a particular focus on the potential applications of deep learning techniques, multimodal data fusion, and remote monitoring systems. This review offers references and support for advancing the development and application of NIFECG monitoring technology.

## 1. Introduction

In recent years, the incidence of congenital heart disease has been increasing, and certain cardiac defects are challenging to detect using conventional ultrasound imaging, underscoring the importance of prenatal fetal cardiac health monitoring. Early methods of fetal monitoring relied on intermittent auscultation of fetal heart sounds. In the 1960s, the invention of electronic fetal monitors accelerated the development of fetal monitoring equipment, which primarily relied on Doppler ultrasound technology [1]. However, clinical practice has revealed that the widespread use of Doppler ultrasound devices is associated with an increase in cesarean section rates and a positive correlation with postpartum depression [2,3].

With the advancement of technology, various fetal monitoring methods have been proposed. For instance, fetal phonocardiography (FPCG) records fetal heart sounds through maternal abdominal sensors, but it is highly susceptible to noise interference and uterine contractions, limiting its long-term monitoring applications [4]. Another method, fetal pulse oximetry monitoring (FPOM), estimates fetal blood oxygen levels based on light absorption differences, enabling the detection of fetal hypoxia. However, FPOM is significantly affected by environmental factors and is primarily applicable only during labor.

NIFECG is a non-invasive fetal monitoring technique that does not emit any signals toward the mother or fetus, making it a truly non-invasive monitoring method. This technique collects abdominal electrocardiography (aECG) by placing electrodes on the maternal abdomen and separating fetal electrocardiography (fECG), maternal electrocardiography (mECG), and noise to enable real-time monitoring of fetal health status [5,6,7]. Compared to other monitoring methods, NIFECG not only provides real-time estimation of the fetal heart rate (FHR) but also offers comprehensive morphological information.

In recent years, fetal monitoring devices based on NIFECG have gradually gained market recognition and regulatory approvals. For example, the Monica Novii Wireless Patch System (2014) and the MERIDIAN M110 fetal monitoring system (2017) have both received FDA approval in the United States.

This paper aims to provide a comprehensive review of the latest research advancements in NIFECG monitoring technology. The structure of this paper is as follows: Section 2 discusses the various stages of the NIFECG monitoring system, including signal acquisition, signal preprocessing, fECG extraction, and fetal cardiac anomaly classification. Section 3 analyzes the performance metrics of existing methods with a focus on the challenges they face. Section 4 discusses and analyzes the existing NIFECG datasets, evaluates their limitations, and proposes suggestions for improvement. Section 5 concludes the paper and presents prospects for future research directions in NIFECG monitoring technology.

## 2. NIFECG Monitoring Technology

**Figure 1:**
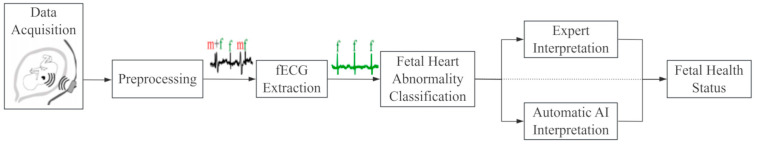
Structure diagram of NIFECG monitoring system.

As shown in Figure 1, the NIFECG monitoring system evaluates fetal health through four main stages: signal acquisition, preprocessing, fECG extraction, and fetal cardiac anomaly classification. First, aECG signals, which include fECG, mECG, and various noise components, are non-invasively collected from the maternal abdomen. After preprocessing, relatively clean mECG and fECG signals are obtained. Subsequently, extraction algorithms are applied to isolate the fECG signal, which serves as input for fetal cardiac anomaly classification to assess fetal health.

Fetal cardiac anomaly classification can be accomplished in two ways: (1) medical experts evaluate fetal health based on FHR interpretation; and (2) intelligent devices perform automatic evaluation. Expert interpretation relies on the FHR patterns. However, due to the absence of unified standards, significant variability may exist among experts. By contrast, intelligent algorithm-based devices overcome the subjectivity and inconsistency associated with manual interpretation. Furthermore, the evaluation results generated by intelligent devices can be directly interpreted by general users, thereby reducing the medical costs to some extent.

This chapter systematically reviews the four main stages of NIFECG monitoring and provides an in-depth discussion of the key technologies involved in each stage.

### 2.1. Signal Acquisition

**Figure 2:**
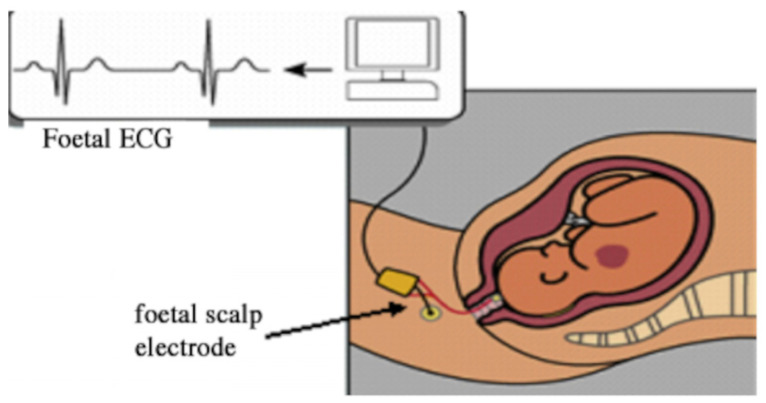
Invasive collection methods. The illustration is sourced from [9].

**Figure 3:**
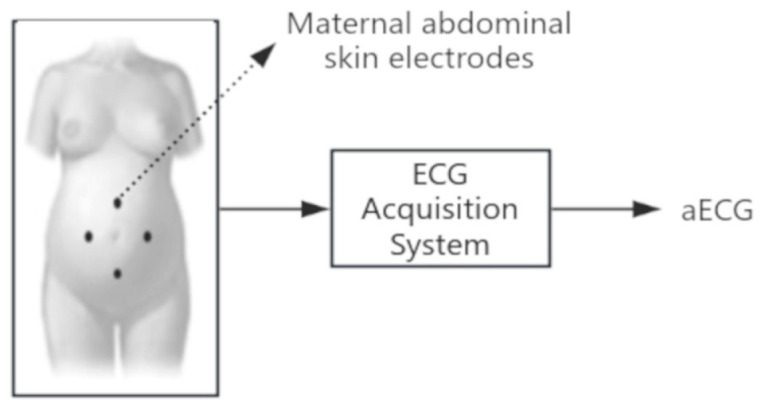
Non-invasive collection methods.

fECG signals can be obtained via invasive or non-invasive methods. Invasive methods (Figure 2) acquire high-quality fECG signals by placing sensors on the fetal scalp. However, due to the risk of infection, this method is only applicable to high-risk pregnancies. In contrast, non-invasive methods (Figure 3) are widely adopted due to their high reliability [8]. Non-invasive acquisitions can be further categorized into single-abdominal and multi-site synchronous acquisitions. Single-abdominal acquisition places sensors on specific abdominal sites to collect aECG, whereas multi-site synchronous acquisition records signals from the abdomen and other regions simultaneously. For instance, Huang et al. [9] collected an ECG signal from the maternal chest. However, factors such as uterine contractions and fetal respiration can make it challenging to identify optimal sensor placement, potentially reducing the signal quality. Additionally, existing sensors often struggle to capture weak signals during early and mid-pregnancy. Therefore, the sensors proposed in References [10,11] provide a new solution for weak signal acquisition. In addition, the selection of collection methods depends on specific application requirements. For example, a single abdominal collection is easy to operate and suitable for scenarios with high portability requirements; multi-part synchronous acquisition uses mECG collected from other parts as a reference, which is suitable for scenarios with high-signal quality requirements. The advantages of single abdominal collection in terms of portability have been demonstrated by Zhang et al. [12], who proposed a single-channel abdominal recording technique for fetal ECG extraction that simplifies the sensor placement process. The benefits of multi-part synchronous acquisition in improving signal quality are highlighted by Mertes et al. [13], where a deep learning approach was used to assess and enhance the signal quality of non-invasive fetal ECG recordings. There are limitations of a single abdominal electrocardiogram signal in certain situations. Some studies have introduced multi-modal physiological signals (such as fetal movement and blood–oxygen saturation) to compensate for the shortcomings of a single signal. Reference [14] obtained electrocardiogram signals through abdominal electrodes and recorded abdominal heart sound signals using a miniature microphone.

### 2.2. Signal Preprocessing

**Figure 4:**
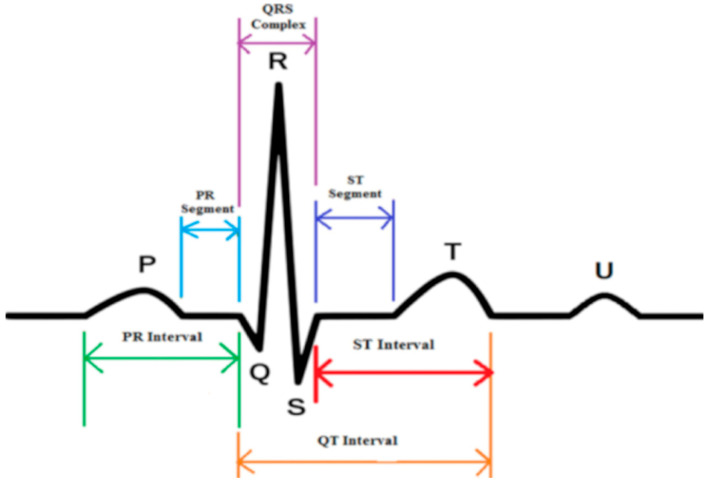
Composition of aECG signal. aECG is a mixture of fECG, mECG, and noise sources. The illustration is sourced from [15].

As shown in Figure 4, non-invasively collected aECG signals exhibit diverse morphological features, such as P-waves and QRS complexes, as well as interval characteristics, including PR intervals and QT durations (Table 1) [15,16]. However, these signals often contain fECG, mECG, and various noise sources (e.g., powerline interference and baseline drift). Therefore, preprocessing, including denoising and ECG segmentation, is essential for accurate analysis.

Traditional denoising methods commonly employ notch, low-pass, and bandpass filters to mitigate noise. For example, broadband notch filters and high–low pass filters have been used to effectively suppress various ECG noises [22,23]. Advances in signal processing have led to the emergence of novel ECG denoising methods that can be categorized into three types based on their technical principles.

1. Signal Decomposition-Based Methods:

Empirical Mode Decomposition (EMD) decomposes ECG signals into Intrinsic Mode Functions (IMFs) and reconstructs them to remove noise [24,25]. However, EMD may suffer from mode-mixing. Singh et al. [26] proposed an ensemble EMD approach that mitigates this issue by introducing white noise and performing multiple decompositions. Wavelet Transform (WT) decomposes signals into multiscale sub-signals for de-noising. For example, Discrete Wavelet Transform (DWT) decomposes a signal into approximation (low-frequency) and detail (high-frequency) coefficients, allowing for the selective filtering of noise. Shahbakhti et al. [27] introduced a mean power frequency (MPF) metric to automatically determine the decomposition levels of DWT, overcoming the need for manual layer selection in traditional DWT. In their approach, the Symlet4 wavelet was used, The MPF is calculated as the mean of the power spectrum of each approximation coefficient, and the decomposition process was stopped when the MPF of the last approximation fell below a specified threshold (e.g., 10 Hz), ensuring that the noise was sufficiently removed while retaining the relevant signal information. In addition, Zahia et al. [28] proposed a hybrid method that combined EMD with wavelet-based mean coefficients. In this approach, the Hurst exponent of noisy IMFs is used to quantify the self-similarity of the signal, and the clean ECG signal is reconstructed by selectively removing noisy IMFs based on their Hurst exponents. Thresholding is crucial in decomposition-based denoising. References [29,30] have employed adaptive techniques, such as instantaneous half-cycle, soft thresholding, and Kullback–Leibler divergence. References [31,32] used polynomial thresholding and bee algorithms to optimize the wavelet coefficients.

2. Deep Learning-Based Denoising Autoencoders (DAE) Methods:

DAE reconstructs signals through an unsupervised learning framework with an encoder–decoder architecture. Chiang et al. [33] demonstrated that, at various signal-to-noise ratios (SNR), DAE based on fully convolutional networks outperforms traditional fully connected networks and Convolutional Neural Networks (CNNs) in denoising. Moham et al. [34] proposed a deep adaptive denoising DAE, which transforms ECG signals into time-frequency images using fractional S-transform for denoising, achieving superior performance in terms of Root Mean Square Error (RMSE).

3. Composite Denoising Methods:

Composite denoising methods refer to the integration of multiple signal-processing strategies, leveraging their individual advantages to achieve more efficient noise suppression. Rakshit et al. [35] combined EMD, wavelet soft thresholding, and adaptive switching mean filter (ASMF). EMD was used to extract IMFs, wavelet soft thresholding addressed noise characteristics at different frequencies, and ASMF was applied for optimization based on varying noise levels. Singh et al. [36] combined variational mode decomposition (VMD), non-local means (NLM) estimation, and DWT to address the sparse effect issue in high-frequency regions.

The advantages and disadvantages of various denoising methods are listed in Table 2.

**Figure 5:**
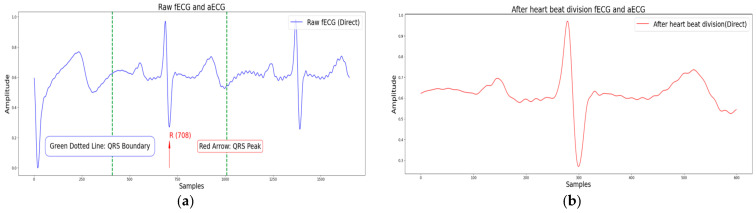
Example of fixed length heartbeat division method. (**a**) Before heartbeat division. (**b**) After heartbeat division.

ECG segmentation refers to the division of ECG signals into individual heartbeats comprising P-waves, QRS complexes, and T-waves. Accurate segmentation is critical for feature extraction and classification. As shown in Figure 5, existing methods typically segment ECG signals using fixed-length windows centered on R-peaks (300 samples per segment). However, such methods fail to account for interindividual variability and environmental influences, leading to incomplete or redundant segments. For this purpose, Reference [38] proposed an adaptive segmentation method that dynamically adjusts the window size and position based on R-wave intervals. This method further normalizes heartbeats with varying durations, ensuring their compatibility with deep learning models.

### 2.3. fECG Extraction

The extraction of NIFECG signals is challenging, primarily because of the significantly weaker intensity of fECG compared with that of mECG and the considerable overlap of their frequency bands. Therefore, efficient algorithms are urgently required to acquire high-quality fECG signals. Based on their technical principles, the existing extraction methods can be categorized into the following four types:

1. Time Domain-Based Methods

**Figure 6:**
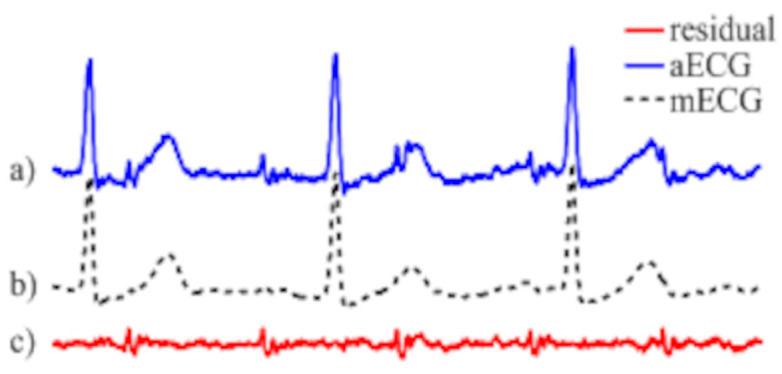
Schematic diagram of the time-domain method. The illustration is sourced from [6].

**Figure 7:**
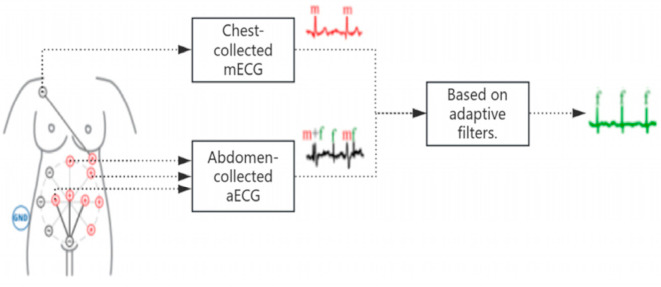
Structure diagram of extracting fECG based on adaptive filter. GND represents ground, + and − are the positive and negative signals, m and f stand for maternal and fetal signals, respectively.

Time domain-based methods utilize the temporal independence between fECG and mECG to generate mECG templates, which are subtracted from aECG to extract fECG (see Figure 6 for an illustration of the template subtraction process). A typical time-domain approach is the EKF-based extraction method, which achieved an accuracy of 95.60%, as demonstrated by Bandi et al. [39]. Karthik et al. [40] proposed a method combining a heuristic recurrent neural network (HRNN) and Kalman filter (KF), achieving an accuracy of 93.118% on the ADFECGDB dataset. Adaptive filters are another commonly used approach for estimating abdominal mECG through an adaptive noise cancelation system and subtracting this estimation from aECG to isolate fECG (see Figure 7 for a schematic representation of the noise cancelation process). These extraction methods can be categorized into adaptive linear and adaptive nonlinear filters. Kahankova et al. [41] developed adaptive linear filters based on LMS and RLS algorithms, which improved fECG quality by optimizing the filter parameters. Nevertheless, filter parameters are influenced by factors such as gestational progress and individual variability, which require dynamic adjustments. To address this, Shadaydeh et al. [42] proposed an adaptive nonlinear Volterra filter (AVF), which models the nonlinear characteristics of maternal thoracic and abdominal signals and integrates all aECG signals as a reference to enhance fECG extraction accuracy. However, the performance of time domain-based methods relies heavily on accurate detection of maternal QRS (mQRS) complexes. When maternal and fetal heartbeats overlap, fECG may be inadvertently removed along with mECG [43,44]. In contrast to traditional time-domain methods, recent advancements have explored time-frequency domain approaches. In [45], a method utilizing short-time Fourier transform (STFT) to generate time-frequency representations of both aECG and fECG was proposed. This approach effectively captures both time and frequency domain features, improving fECG extraction accuracy even in the presence of significant maternal heart interference. The proposed model, incorporating a hierarchical multi-scale perceptual network, achieved a Pearson correlation coefficient (PCC) of 0.845 on the PhysioNet dataset.

2. Spatial Domain-Based Methods

Spatial domain-based methods exploit the spatial distribution characteristics of source signals and utilize the spatial information recorded by sensors at different positions to achieve the separation of fECG, mECG, and noise, thereby extracting the fECG signal. Independent component analysis (ICA) is one of the most commonly used signal separation techniques in this domain. However, ICA has limitations in identifying the number, order, and amplitude of the signals. To address this, Ref. [7] proposed optimizing the initial weights of FastICA by combining it with over-relaxation factors and Newton’s iteration algorithm, thereby enhancing convergence performance. Ziani et al. [46] were the first to apply a method that integrates wavelet transform, singular value decomposition (SVD), and ICA for fECG extraction. This approach leverages the wavelet transform and SVD to extract time-frequency information and compress energy components from the signal before applying ICA to extract fECG. However, its performance on the DAISY dataset indicated substantial room for improvement.

3. Wavelet Transform-Based Methods

In aECG, fECG and mECG generally exhibit distinct frequency components, making WT an effective tool for separating these signals. Jallouli et al. [47] achieved 100% extraction sensitivity on the DAISY and PhysioNet datasets by combining multi-wavelet methods with Shannon entropy evaluation algorithms. The method employed multi-wavelet approaches, where both forward and inverse wavelet transforms were performed using 5-level decomposition. The Shannon entropy algorithm was applied to optimize the wavelet coefficients, and thresholding was performed using a soft-thresholding technique. Darsana et al. [48] utilized recursive least squares (RLS), stationary wavelet transform (SWT), and an improved spatially selective noise filtering approach to extract fECG signals. In this approach, SWT was applied using the Bior 1.5 wavelet, a member of the wavelet family, with five-level decomposition. Subsequently, RLS was used to optimize the wavelet coefficients. The RLS algorithm was implemented with a step size of 0.1, and the forgetting factor was set to 0.99. A spatially selective noise filtering method was employed to remove noise from the signal, and the noise threshold was determined by calculating the error between the noisy signal and original wavelet detail coefficients. The accuracies achieved were 97.01% and 94.35% for the DAISY and NIFECGDB datasets, respectively. However, WT methods may introduce signal distortion during processing, making them more suitable for FHR calculations than for morphological analysis.

4. Deep Learning-Based Methods

Deep learning-based methods have demonstrated remarkable performance in recent years. Common methods include the following:

- Adaptive neuro-fuzzy inference system (ANFIS): ANFIS combines neural networks with fuzzy inference systems, showing outstanding performance in fECG extraction [49,50]. S. H. Jothi et al. [50] proposed combining ANFIS with undecimated wavelet transform (UWT), where UWT removes noise, and ANFIS models the nonlinear relationship between mECG and aECG to extract fECG.
- Long short-term memory (LSTM)-based: LSTM excels at capturing long-term dependencies in sequential data. Zhou et al. [51] introduced a two-stage slow-fast LSTM (SFLSTM) model, where the first stage uses slow LSTM to remove mECG and noise, and the second stage employs fast LSTM to enhance the fECG signal. Compared to conventional LSTM, SFLSTM not only improved accuracy but also reduced computational costs by approximately 50%. Darmawahyuni et al. [52] achieved 100% accuracy in fetal QRS detection by combining CNN and BiLSTM.
- Transformer-based architectures: Transformers’ positional encoding and self-attention mechanisms offer advantages for extracting high-quality fECG and morphological analysis. Chen et al. [53] proposed a model combining CNN and transformer to extract and detect R-peaks in fECG signals, achieving an F1-Score exceeding 98%. M. Almadani et al. [54] proposed a novel transformer-based architecture, referred to as W-NET transformers (W-NETR), for NIFECG extraction. The W-NETR model integrates U-Net and transformer layers, facilitating the simultaneous reconstruction of both fECG and mECG signals. The results indicated that the F1-Score of this approach was 99.88% on the ADFECGDB dataset.
- Generative adversarial networks (GANs)-based: GANs are a class of deep learning frameworks consisting of a generator and a discriminator that are optimized through adversarial training. P. Basak et al. [55] proposed a method utilizing 1D CycleGAN to reconstruct fECG signals from mECG data while preserving their morphological features. By incorporating spectral loss, temporal loss, and power loss functions, the method enhances the quality of the extracted signals. On the PhysioNet dataset, this approach achieved an F1-Score of 96.4%, Pearson correlation coefficient (PCC) of 88.4%, and spectral correlation score of 89.4%. Moreover, the model achieved low errors of 0.25% and 0.27% in fetal heart rate and R-R interval estimations, respectively.

Overall, spatial domain-based methods excel in extracting additional cardiac information, such as cardiac hypercontraction, but typically require a large number of abdominal electrodes, which may cause discomfort in pregnant women. In contrast, time domain-based methods are simpler to implement and more practical for clinical applications [56]. Moreover, most existing methods require dynamic adjustments based on factors such as fetal position, gestational progress, and electrode placement. Deep learning-based approaches can automatically extract complex features and dynamically adjust model parameters, showing strong potential in ECG-related tasks such as signal denoising, feature extraction, and classification. Although the aforementioned algorithms show promise, differences in datasets and accuracy definitions across evaluations hinder definitive conclusions. Therefore, the performance of new techniques should be validated under standardized datasets and evaluation criteria.

### 2.4. Fetal Cardiac Abnormality Classification

Fetal cardiac abnormalities may pose a significant threat to fetal survival. The accurate classification of these abnormalities is crucial for identifying potential health issues and reducing mortality risks. Based on ECG signal characteristics, experts classify fetal health status into 29 multi-label cardiac abnormalities [57]. According to the standards set by the American Association for Medical Instrumentation, these abnormalities are categorized into five types: normal, supraventricular ectopic beats, ventricular ectopic beats, fusion beats, and unknown [58].

**Figure 8:**
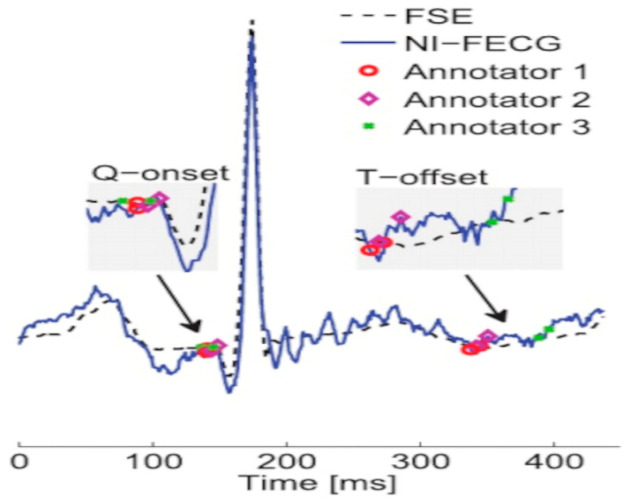
Annotations by three experts on fetal scalp electrocardiogram (FSE) and NI-FECG waveforms. Each expert independently marked two points: the onset of the Q-wave and end of the T-wave. The illustration is sourced from Ref. [62].

The classification of fetal cardiac abnormalities primarily relies on ECG features such as FHR and morphological parameters. These features reflect fetal cardiac electrical activity and are used to distinguish between normal and abnormal cardiac states. During classification, the annotation accuracy is of paramount importance. As illustrated in Figure 8, annotations are derived from expert manual interpretations, which often suffer from inconsistency. It is recommended that data annotations be obtained from at least three experts by consensus [59,60,61,62].

Promising methods for fetal cardiac abnormality classification can be broadly divided into two categories: traditional machine learning-based algorithms and deep learning-based algorithms.

Traditional machine learning approaches rely on manual feature engineering, which typically requires less training data and achieves high classification accuracy in certain scenarios. Common methods include the following:

- Support vector machine (SVM)-based methods: Saini et al. [63] employed SVM to classify QRS complexes, P-waves, and T-waves, thereby differentiating normal and abnormal waveforms. Pavel et al. [64] developed a Gaussian kernel-based SVM classifier using morphological features to achieve fetal abnormality classification, with a classification accuracy of 83.33%.
- Bayesian classifiers: Apsana et al. [65] utilized a Naïve Bayes classifier, assuming independence among features, to achieve a classification accuracy of 93.71% in fetal cardiac abnormality detection.

Deep learning methods reduce reliance on manual feature engineering by automatically extracting features, thereby demonstrating superior classification performance. These methods include the following:

- CNN- and LSTM-based classification methods: CNN-based approaches can be categorized into 1D and 2D CNN, depending on the input signal type. One-dimensional CNN is suitable for raw or denoised ECG signal classification. Jin et al. [66] employed 1D CNN for the classification of various cardiac conditions, incorporating few-shot learning for small datasets. The 2D CNN primarily handles image-like inputs such as ECG spectrograms and wavelet plots. In [67], ECG signals were transformed into 2D wavelet plots and classified using AlexNet. LSTM leverages the inherent temporal correlations in the ECG and effectively captures category-specific features. Zhou et al. [68] designed a six-layer LSTM model for automatic identification of ventricular premature beats in ECG signals. Xu et al. [69] adopted a bidirectional LSTM (BiLSTM) for temporal ECG signal classification.
- Encoder–decoder architecture-based methods: Transformer, as an encoder–decoder architecture, utilizes a self-attention mechanism to effectively assign weights to features [70]. Yan et al. [71] applied the encoder component of Transformer for cardiac abnormality classification in ECG signals. Akan et al. [72] presented ECGformer, a Transformer model optimized specifically for ECG signals, achieving 97% classification accuracy in multi-class classification. Additionally, convolutional autoencoders (CAE), a variant of autoencoders, do not require labeled data and are particularly suitable for tasks with scarce annotations. Arslan et al. [73] proposed a CAE capable of effective training with limited labeled data, thereby reducing annotation costs.

In summary, traditional machine learning methods perform robustly on small datasets, but fail to fully exploit morphological features, limiting their efficacy in complex cardiac abnormality classification tasks. In contrast, deep learning-based approaches have demonstrated outstanding performance in the classification of fetal cardiac abnormalities. However, data imbalance remains a significant challenge for deep learning models. Because abnormal data constitutes only a small fraction of the total dataset, models tend to favor majority classes, potentially leading to misleading accuracy metrics.

## 3. Performance Metrics Analysis

This chapter comprehensively evaluates the performance of fECG extraction and fetal cardiac anomaly classification tasks. These evaluation methods cover several key metrics, including R-wave detection, morphological analysis, and various indicators for classification tasks [41,74]. Based on the definitions of True Positive (TP)—the number of correctly identified positive instances; False Positive (FP)—the number of negative instances incorrectly predicted as positive; True Negative (TN)—the number of correctly identified negative instances; and False Negative (FN)—the number of positive instances incorrectly predicted as negative, the following performance metrics are adopted:

- Sensitivity (Sen):

(1) $$Sen=\frac{TP}{TP+FN}$$

- Positive predictive value (PPV):

(2) $$PPV=\frac{TP}{TP+FP}$$

- Accuracy (Acc):

(3) $$Acc=\frac{TP}{TP+FP+FN}$$

- F1-Score:

(4) $$F1\text{-}Score=2\times \frac{PPV\times Sen}{PPV+Sen}$$

These metrics are widely used in both fECG extraction and fetal cardiac anomaly classification tasks. Although their calculation formulas are the same, their specific meanings vary for different tasks.

### 3.1. fECG Extraction Performance Evaluation

**Figure 9:**
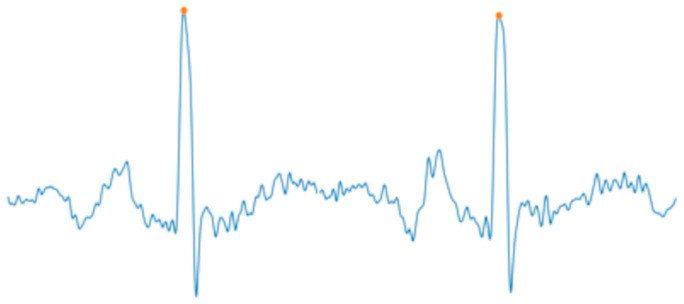
Example of detecting fQRS peaks, with orange dots representing the positions of the detected fQRS peaks.

The performance evaluation of the fECG extraction task primarily focuses on the detection accuracy of fetal QRS (fQRS) complexes and signal quality. For fQRS peak detection, according to ANSI/AAMI standards, valid detection results require that the distance between the detected peak and reference annotation point falls within ±50 ms. Therefore, a ±50 ms window is typically used to assess fQRS detection results. Common metrics, such as Sen, PPV, Acc, and F1-Score, are employed to evaluate fQRS detection accuracy. Specifically, Sen represents the proportion of correctly detected fQRS peaks relative to the total number of reference fQRS peaks; PPV indicates the proportion of correctly detected fQRS peaks relative to all detected fQRS peaks; Acc reflects the proportion of correctly detected fQRS peaks relative to all detected fQRS peaks; and F1-Score is the harmonic mean of Sen and PPV. Figure 9 illustrates an example of QRS peaks detection in fECG signals. In addition, the RMSE is calculated to measure the discrepancy between reference fQRS peaks and detected fQRS peaks, as expressed by the following formula:

(5) $$RMSE=\sqrt{\frac{1}{N}\sum_{i=1}^{N}{\left(r_{i}-f_{i}\right)}^{2}}$$

where $r_{i}$ and $f_{i}$ represent the reference and detected fQRS peak positions, respectively.

Morphological analysis is also a critical approach for evaluating the quality of extracted fECG signals. A commonly used metric is SNR, which is defined as:

(6) $$SNR=20\log_{10}\frac{\sum_{i=1}^{N}r_{i}^{2}}{\sum_{i=1}^{N}{{(r}_{i}-f_{i})}^{2}}$$

where $r_{i}$ and $f_{i}$ represent the reference and extracted signals, respectively.

However, SNR provides only an overall assessment of the extracted signal quality and does not quantify whether clinically important parameters are preserved in the extracted NIFECG. Therefore, a combined evaluation of morphological features and SNR is essential for assessing the quality of fECG extraction [61].

### 3.2. Evaluation of Fetal Cardiac Anomaly Classification Performance

The fetal cardiac anomaly classification task is typically regarded as a time-series classification problem, with evaluation metrics including Sen, PPV, Acc, and F1-Score. Although the calculation formulas for these metrics are identical to those used in the extraction tasks, their definitions and applications differ. Specifically, Sen represents the proportion of correctly identified positive samples relative to all actual positive samples; PPV indicates the proportion of actual positive samples among those classified as positive; Acc reflects the proportion of correctly classified samples among all samples; and F1-Score is the harmonic mean of Sen and PPV. The specific formulas are shown in Equations (1)–(4).

### 3.3. Performance Metric Analysis Under Class Imbalance

There is a significant class imbalance in ECG datasets, with abnormal samples accounting for a small proportion. This imbalance may lead to confirmation bias, causing evaluation metrics to show falsely good performance. To avoid misguidance, the following strategies are recommended:

- Adopt F1-Score as the primary performance metric: In class-imbalanced scenarios, the model may produce a high number of false positives (negative samples misclassified as positive). By balancing Sen and PPV, the F1-Score effectively penalizes these false positives, making it particularly suitable for addressing class-imbalanced datasets [75,76,77,78].
- Use Sen and correlation coefficients to enhance comparability: Sen complements the F1-Score in class-imbalanced conditions, providing a more comprehensive evaluation. Correlation coefficients capture discrepancies between predicted and true values, ensuring accurate performance assessment despite uneven class distributions [77,78].
- Avoid relying solely on Acc: In class-imbalanced datasets, accuracy may result in falsely high scores due to the large proportion of negative samples. This should be avoided in the performance evaluation.
- Visualize sample results: Intuitive visual comparisons should be used for visual evaluation to avoid statistical errors.

## 4. Discussion and Analysis of NIFECG Datasets

Before the 2013 PhysioNet Challenge, commonly used datasets included the following:

- **DAISY (1997)** comprises eight ECG channels from a single fetus (three thoracic and five abdominal channels) with 10 s of recordings sampled at 250 Hz and no reference annotations. However, the generalizability of DAISY is limited, as it includes data from a single fetus and has a relatively short duration, which reduces its applicability to more diverse fetal monitoring scenarios [79].
- **NIFECGDB (2000)** consists of recordings from a single pregnant subject (gestational weeks 21–40), including 55 multi-channel aECG recordings (two thoracic and three or four abdominal channels) sampled at 1 kHz without reference annotations. While this dataset provides more comprehensive coverage of pregnancy stages, it still lacks diversity in both the number of subjects and the presence of reference annotations, which limits its utility in training and validating robust fetal ECG extraction models [80].
- **ADFECGDB (2000)** includes recordings from five laboring pregnant subjects (gestational weeks 38–41), with each recording containing four abdominal and one fetal scalp channels. The duration of each recording was 5 min, with QRS complexes annotated in fetal scalp ECG signals. However, the small sample size and limited fetal scalp data may not fully represent the diversity of fetal heart rate abnormalities or complex maternal–fetal interactions in different stages of pregnancy [81].

In 2013, PhysioNet released the largest publicly available fECG dataset, comprising 447 min of data sourced from multiple datasets (e.g., NIFECGDB, ADFECGDB, and synthetic data generated by fECGSYN). All data were resampled to 1 kHz, with each recording containing four abdominal channels and no maternal reference signal. While some datasets include QRS annotations, others restrict access to these annotations to mitigate the risk of overfitting during algorithm development [82]. This challenge highlights the demand for diverse datasets in the NIFECG domain, prompting the development of several new datasets, including the following:

- **fECGSYNDB (2016)** contains simulated signals from 10 different pregnant subjects recorded under five noise levels, ten maternal-fetal dipole arrangements (e.g., different maternal and fetal positions and relative orientations), and seven physiological scenarios. It includes 32 abdominal and 2 thoracic channels, resulting in 1750 simulated abdominal signals, each with a duration of 5 min and sampled at 1 kHz. However, due to its purely synthetic nature, this dataset may not fully capture the complexities and variability encountered in real-world clinical settings, which may limit its generalizability and applicability in practical applications [83].
- **NIFEADB (2019)** included recordings from 26 fetuses, 12 with arrhythmias, and 14 normal fetuses. Each recording contains four or five abdominal channels and one thoracic channel. The recordings from arrhythmic fetuses had a duration of 13 min and 3 s, whereas those from normal fetuses lasted 10 min and 6 s, sampled at either 500 Hz or 1 kHz [84].

Table 3 summarizes the six publicly available NIFECG datasets. The use of the most recent and comprehensive real-world datasets should be prioritized to ensure the robustness and generalizability of the developed models.

Real NIFECG datasets are limited by the lack of pathological cases (e.g., complications such as adverse pregnancies or rare events like maternal-fetal heart rate similarity) and are insufficient for assessing algorithm performance under varying signal qualities. This limitation has driven the development of simulated NIFECG models. The fECGSYNDB dataset, for instance, provides simulated data that facilitates the modeling of physiological phenomena under controlled conditions. However, real ECG waveforms should adhere to specific physiological intervals, while simulated data, often derived from linearly scaled adult ECG waveforms, may not conform to these physiological norms. Additionally, fECGSYNDB does not yet model the relationship between morphological features and FHR, necessitating derivation and correction from real data.

To address these challenges, this study proposes two strategies: data augmentation and construction of new datasets.

**Figure 10:**
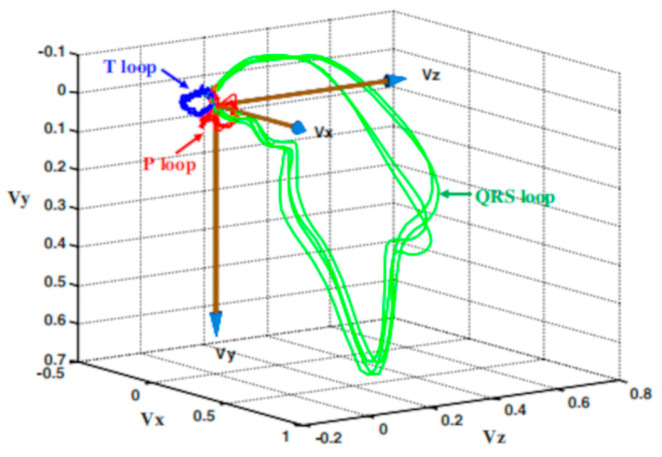
VCG Loop. The largest green QRS loop represents ventricular depolarization. The red P-wave indicates atrial depolarization, whereas the blue T-wave indicates ventricular repolarization. Figure adapted from [91].

Data augmentation, an effective technique for expanding training datasets, has been widely applied and can be broadly categorized into perturbation-based data augmentation and synthetic data generation. Perturbation-based data augmentation generates new data by applying transformations such as scaling, translation [85], or adding noise [86] to ECG signals, producing samples that are highly correlated with the original data. Synthetic data generation creates new samples by linearly combining real data or simulating ECG features. Common methods include SMOTE and its variants such as Borderline-SMOTE [87] and SVM-SMOTE [88,89] which alleviate data imbalance problems through sample interpolation or boundary sample expansion. Vectorcardiography (VCG) is another significant approach to synthetic data generation. By reconstructing the three-dimensional vector loops of cardiac electrical activity from three-lead ECG signals, VCG intuitively visualizes the spatial distribution characteristics of cardiac electrical activity. This is achieved by plotting vector loops that represent the electrical activity of the heart in a three-dimensional space (see Figure 10) [90,91]. These vector loops provide a visual representation of the heart’s electrical behavior during each cardiac cycle. The analysis of these loops can reveal important spatial-temporal characteristics, such as the direction and magnitude of the heart’s electrical activity, helping to simulate synthetic ECG data with specific pathological features. Reference [91] further introduced spatiotemporal dynamic analysis techniques, such as velocity, phase angle, and curvature changes in the vector loops, to capture the spatiotemporal variations in ECG signals, enabling the simulation of synthetic ECG data with specific pathological features. In recent years, deep learning has demonstrated significant potential in the field of data augmentation. For instance, GANs [92,93] and GAN variants based on Variational Autoencoders (VAEs) [94] can generate high-quality and diverse synthetic ECG data.

The PhysioNet dataset, among others, has established a standard for constructing new datasets: data should encompass physiological information, including abdominal, fetal, and gestational signals, with detailed annotations, such as physiological timing and labels. Although these datasets have laid the foundation for the development and evaluation of NIFECG algorithms, they still exhibit limitations in terms of diversity, data volume, and coverage of pathological cases. Therefore, it is imperative to construct a dataset that includes multi-channel signals and pathological cases. The desired dataset should possess the following characteristics:

- This should include signals recorded from abdominal and thoracic channels. For pregnant women during labor, scalp channel recordings are required to serve as a basis for the morphological analysis of NIFECG signals;
- The signal duration should be at least 5 min, ideally exceeding 30 min, with a sampling frequency no less than 1 kHz;
- The dataset should document the health status of both the mother and fetus to facilitate research on intelligent monitoring devices.

## 5. Conclusions and Future Directions

In recent years, NIFECG monitoring technology has made significant progress, particularly in signal preprocessing, extraction, and classification algorithms. However, the existing technologies still face numerous challenges that require further investigation. Specifically, while invasive scalp ECG has been widely used in morphological analysis, the morphological analysis of NIFECG remains in its infancy, limiting its clinical diagnostic applications to some extent. Moreover, the acquisition of high-quality signals imposes higher demands on sensor placement and algorithm performance.

To establish NIFECG as a standard fetal monitoring technology, future research can focus on the following key directions:

1. Deep Learning-Based NIFECG Monitoring Methods

Deep learning has demonstrated great potential in NIFECG monitoring, particularly for tasks such as ECG denoising, feature extraction, and classification. The research priorities include the following:

- Model architecture optimization: Considering the characteristics of ECG data, model architectures should be designed to capture signal periodicity and trends. For example, introducing positional encoding [95] and periodic features can enhance model performance. In addition, neural architecture search (NAS) techniques can be employed to automate the optimization of model structures and identify the optimal combination of feature extraction and classification strategies [96,97].
- Personalized medical model design: Given the individual physiological differences between mothers and fetuses, deep learning models capable of dynamically adjusting parameters should be developed. These models should be able to self-optimize as the data volume increases, thereby meeting the needs for long-term, personalized monitoring [98,99].

2. Multi-Modal Fusion Based on NIFECG

Multimodal data fusion [100] combines various physiological signals (e.g., uterine activity [83], fetal movement [101,102], and maternal posture [103]), providing more comprehensive information for clinical diagnosis. However, a major challenge in multimodal fusion is efficient integration of diverse physiological signals. Therefore, the development of innovative multimodal fusion algorithms is critical. For instance, attention mechanism-based fusion algorithms can dynamically allocate weights to each modality based on specific task requirements, thereby enhancing the robustness of the fusion outcomes. Recent studies have demonstrated the potential of multimodal fusion in improving NIFECG monitoring. For example, integrating uterine activity with fetal heart rate has shown to improve labor prediction accuracy [102], and combining fetal movement data with ECG signals enhances detection of fetal distress [101].

3. Remote Fetal ECG Monitoring Systems and Portable Devices

Remote fetal ECG monitoring systems integrate edge computing and wireless communication technologies (e.g., 5G and Wi-Fi) to enable the real-time acquisition and transmission of fetal ECG data. This allows healthcare providers to dynamically monitor fetal health in remote settings, ensuring maternal and fetal safety [104,105,106,107]. Such technology is particularly valuable for managing high-risk pregnancies and providing medical care in resource-limited, remote areas; the use of wireless NIFECG monitoring in rural India [108] demonstrated the feasibility and impact of remote monitoring in improving maternal and fetal health management. Furthermore, the development of wearable and portable devices offers effective solutions for offline NIFECG monitoring. By leveraging edge computing, data processing models can be deployed on low-power, wearable smart devices (e.g., smart abdominal belts, smart clothing), enabling localized data analysis and improving the real-time performance and efficiency of monitoring [108,109]. These intelligent devices also support long-term continuous monitoring, offering promising potential to advance the clinical applications of remote fetal ECG monitoring systems. However, deploying deep learning models on portable devices and in low-resource settings remains challenging due to computational and memory constraints. Techniques such as model compression and edge computing are crucial for addressing these limitations and enabling efficient real-time monitoring in such environments.

## Figures and Tables

**Table 1:** ECG morphological parameters and time interval definitions.

Morphological Parameter Name	Definition	Time Interval
P-wave	The duration from the beginning to the end of P-wave.	22–83 ms ([17])
QRS complex	The duration from the beginning of Q-wave to the end of S-wave.	18–75 ms ([17]), 47–85 ms ([18])
T-wave	The duration from the beginning to the end of T-wave.	85–180 ms ([17])
PR section	The duration between the end of P-wave and R-wave.	66–166 ms ([17])
PR interval	The duration between the onset of the P-wave and the onset of the QRS complex.	120–200 ms ([19,20])
QT interval	The duration from the beginning of Q-wave to the end of T-wave.	350–450 ms ([20,21])

**Table 2:** Analysis of the advantages and disadvantages of different denoising methods.

Denoiseing Method	Advantages	Disadvantages
Signal Decomposition-Based Methods	Highly effective in extracting ECG signal features, suitable for complex noise environments [37]. Capability for denoising across different time-frequency scales [27,29].	Mode-mixing issues in EMD [30]. Complexity in threshold selection [27,31].
Deep Learning-Based DAE Methods	Feature captures via nonlinear structures, adaptable to diverse noise [33,34]. Adaptive learning capability.	Dependence on high-quality labeled data for training [34]. High computational resource demand for model training.
Composite Denoising Methods	Integration of multiple denoising techniques, adaptable to various noise types. Suitability for complex and dynamic environments.	Complexity in system design due to integration of different techniques. Time-consuming and less efficient optimization process.

**Table 3:** Feature summary of publicly available NIFECG datasets. Adapted from [6].

Datasets	Duration	Sampling Frequency in Hz	Number of Recordings	Number of Signals	QRS Annotations	Number of Fetuses
DAISY	10 s	250	1	3 mECGs 5 aECGs	no	1
NIFECGDB	1.9 to 46.3 min	1000	55	2 mECGs 4 aECGs	no	1
ADFECGDB	5 min	1000	5	4 aECGs 1 fECG	yes	5
PhysioNet training set	1 min	1000	75	4 aECGs	yes	unknown
PhysioNet val and test set	1 min	1000	100	4 aECGs	no	unknown
fECGSYNDB	1 min	1000	5	4 aECGs 1 mECG	yes	10
NIFEADB	10.1 min and 13.05 min	500 or 1000	26	4 or 5 aECGs 1 mECG	no	26

## Data Availability

The data presented in this study are publicly available in the DAISY and PhysioNet, reference number [82,83,84,85,86,87].
